# Associations of serum uric acid and urinary albumin with the severity of diabetic retinopathy in individuals with type 2 diabetes

**DOI:** 10.1186/s12886-020-01713-5

**Published:** 2020-11-30

**Authors:** Donghe Chen, Xiufang Sun, Xinxin Zhao, Ying Liu

**Affiliations:** 1 Department of Endocrinology, Daqing People’s Hospital, the Fifth Affiliated Hospital of Harbin Medical University, No.213, Jianshe Road, Development zone, Daqing, 163000 China; 2 Department of Endocrinology, the First Affiliated Hospital of Qiqihar Medical University, Qiqihar, 161041 China; 3 Department of Endocrinology, the First Hospital of Suihua, No. 180 Beilin Road, Beilin District, Suihua, 152000 China

**Keywords:** Type 2 diabetes mellitus, Diabetic retinopathy, Serum uric acid, Urinary albumin

## Abstract

**Background:**

Diabetic retinopathy (DR) is a serious microvascular complication of type 2 diabetes mellitus (T2DM). The aim of this retrospective study was to reveal the risk factors for the severity of DR in individuals with T2DM. Demographic data and biochemical parameters were collected and analyzed.

**Methods:**

A total of 518 individuals with type 2 diabetes were included. These individuals were classified into three groups according to the severity of diabetic retinopathy: non-diabetic retinopathy (NDR) group (*N* = 172), non proliferative diabetic retinopathy (NPDR) group (*N* = 184), and proliferative diabetic retinopathy (PDR) group (*N* = 162). Demographic and clinical measurement data of the individuals were collected by reviewing medical records and direct interview. The demographic data and biochemical parameters between groups were compared using Student’s t-test. Moreover, the factors related to severity of diabetic retinopathy were identified by using the multivariate logistic regression analysis.

**Results:**

No significant difference in age, gender, body mass index (BMI), and diabetes duration was found among these three groups. The serum uric acid (SUA), total cholesterol (TC), low density lipoprotein cholesterol (LDL-c), homocysteine, and urinary albumin levels were significantly higher in the NPDR and PDR group than those in the NDR group (*P* < 0.05). The individuals in the PDR group had obviously higher levels of SUA, homocysteine, and urinary albumin than individuals in the NPDR group (*P* < 0.05). The multivariate logistic regression analysis revealed that high SUA, homocysteine, TC, LDL-c, and urinary albumin levels were associated with more serious diabetic retinopathy (OR > 1; *P* < 0.05).

**Conclusion:**

The concentrations of SUA and urinary albumin are associated with the severity of DR in individuals with T2DM.

## Background

Diabetes mellitus is the most prevalent metabolic disease with the characteristic of hyperglycaemia, and it is caused by defects in the endogenous insulin secretion or action [1]. It has been estimated that the number of people with diabetes aged 20–79 years would rise to about 642 million by 2040 which increased 62% compared to 2015 [2]. Type 2 diabetes mellitus (T2DM), accounting for approximately 90–95% of diabetes, is an expanding global health problem [1, 3]. T2DM has high risk of causing macrovascular complications (i.e. cardiovascular disease) and microvascular complications (such as diabetic retinopathy, diabetic nephropathy and diabetic neuropathy) because of hyperglycaemia and insulin resistance syndrome [4].

Diabetic retinopathy (DR) is triggered by hyperglycemia which causes increased oxidative stress leading to an adaptive inflammatory assault to the neuroretinal tissue and microvasculature [5]. Clinically, DR could be divided into background and proliferative stage according to the severity [6]. The people with background stage DR had lesions on the eye vasculature layer and vision lost would be caused if there is fluid in the central portion of the eyes [7]. The proliferative DR (PDR) is characterized by pathological retinal neovascularization and retinal vascular leakage (macular edema) [8]. DR is the most frequent cause of new cases of blindness among adults aged 20–74 years, resulting in central vision loss caused by microvascular damage to the retina [9]. If DR is diagnosed early, effective treatment could delay its onset and progression. Therefore, identification of the clinical markers associated with severity of DR might be beneficial for the early detection and management of DR.

Serum uric acid (SUA), the final oxidation product of purine metabolism in circulation, and many studies have revealed that elevated SUA levels are positively associated with the development of metabolic diseases (such as hypertension, chronic kidney disease, and T2DM) [10–12]. An elevated SUA level could be used as a strong and independent risk factor for renal function decline in individuals with T2DM [13]. Albuminuria is known as one of the risk factors of renal disease since the kidneys normally do not filter large molecules into the urine [14]. Microalbuminuria has been reported to be an independent predictor of severity of coronary artery stenosis in individuals with T2DM [15]. However, whether the SUA and urinary albumin are associated with the severity of DR is still controversial. In our study, the included individuals with T2DM were classified into three groups according to the severity of diabetic retinopathy. The demographic data and biochemical parameters among the three groups were compared using statistical analysis. Moreover, the risk factors for severity of DR were identified by using the multivariate logistic regression analysis.

## Methods

### Population

The type 2 DM was diagnosed by detecting the fasting blood glucose level based on the diagnostic criteria published by World Health Organization [16]. Based on the detection results, individuals diagnosed as T2DM and hospitalized in endocrine department of our hospital from 2015 to 2017 were firstly included in our study. Afterwards, individuals with hypertension, coronary heart disease, severe pulmonary disease, kidney disease, liver disease, severe infection, tumor, or have recently taken the drugs affecting uric acid level (such as diuretics, hydrochloric acid drugs, hyaluronic acid drugs, sodium bicarbonate, etc.) were excluded. Finally, 518 people were included according to the above inclusion and exclusion criteria.

Demographic data of the individuals were collected by reviewing medical records and direct interview, which included age, gender, body mass index (BMI), and diabetes duration. The study was approved by Institutional Human Ethical Committee. Written informed consents were obtained from all the enrolled individuals.

### Assessment of diabetic retinopathy

The enrolled people with T2DM were divided into three groups, including non-diabetic retinopathy (NDR) group, non proliferative diabetic retinopathy (NPDR) group, and proliferative diabetic retinopathy (PDR) group, by two experienced retina specialists according to grading standards published by the Early Treatment Diabetic Retinopathy Study (ETDRS) Research Group based on the results of fundus photographs and fundus fluorescein angiography [17].

### Biochemical measurements

The venous blood samples were collected from each individual in the morning after fasting for 8–12 h. All individuals accepted the routine measurements, including HbA1c and lipid profile, including total cholesterol (TC) and low density lipoprotein cholesterol (LDL-c). Serum HbA1c concentration was measured with the Direct Enzymatic HbA1c Assay Kit purchased from the Shanghai Rongsheng Biological Pharmaceutical Co. (Shanghai, China) by using an automatic glycohemoglobin analyzer (HA-8180, Arkray, Inc., Japan). The levels of LDL-c and TC were measured by commercially available assay kits (Beijing Labo Biotech Co., Ltd., China) with Hitachi 7600 Automatic Biochemical Analyzer (Hitachi Co., Japan).

Moreover, serum uric acid (SUA) was measured by the commercially available assay kits (Shanghai Kehua Bio-engineering Co. Ltd., China) using the Hitachi 7600 Automatic Biochemical Analyzer (Hitachi Co., Japan). The homocysteine level was detected by enzymatic cycling assay kits (NingBo MedicalSystem Biological Technology Co., Ltd., China) with the Hitachi 7600 Automatic Biochemical Analyzer. The microalbuminuria was detected by immunoturbidimetry assay kits (Beijing Leadman Biochemistry Co., Ltd., China) with Hitachi 7600 Automatic Biochemical Analyzer. The definition of microalbuminuria is urinary albumin excretion level > 30 mg/mL.

### Statistical analysis

Data were analyzed using SPSS statistical software (Chicago, USA). Data were expressed as mean ± SD. Student’s t-test was used to evaluate the significances between the groups in age, gender, BMI, diabetes duration, SUA, homocysteine, TC, LDL-c and urinary albumin levels. Multivariate logistic regression was employed to evaluate the associations of SUA, homocysteine, TC, LDL-c and urinary albumin levels with severity of DR. A value of *p* < 0.05 was considered as statistically significant.

## Results

### Demographic data

This study enrolled 518 individuals, including 278 male (54%) and 240 female (46%) individuals with type 2 DM. These individuals with diabetes were classified into NDR group (*n* = 172), NPDR group (*n* = 184) and PDR group (*n* = 162) according to the severity of diabetic retinopathy. There was no significant difference in age, gender, BMI, and diabetes duration among these three groups (Table 1).

**Table 1:** Demographic characteristics of people with diabetes in the non-diabetic retinopathy (NDR), non proliferative diabetic retinopathy (NPDR) and proliferative diabetic retinopathy (PDR) group

Characteristics	NDR group (*N* = 172)	NPDR group (*N* = 184)	PDR group (*N* = 162)
Age (years)	49.2 ± 8.5	52.1 ± 13.1	53.5 ± 10.1
Male/Female	88/84	96/88	84/78
BMI (kg/m^2^)	23.1 ± 1.6	21.9 ± 2.8	24.6 ± 3.6
Diabetes duration (years)	10.5 ± 1.7	8.39 ± 3.9	9.4 ± 2.6
HbA1c	6.3 ± 1.4	7.1 ± 2.8	7.2 ± 3.2

*BMI* body mass index, *HbA1c* glycated hemoglobin A1c

### Comparison of clinical characteristics

The clinical characteristics of the individuals with diabetes in the three groups are summarized in Table 2. Compared with the individuals with diabetes in the NDR group, the SUA, homocysteine, TC, LDL-c and urinary albumin levels of individuals with diabetes in the NPDR and PDR group were significantly higher (*P* < 0.05). The SUA, homocysteine, and urinary albumin levels of individuals with diabetes in the PDR group were obviously higher than those of individuals with diabetes in the NPDR group (*P* < 0.05).

**Table 2:** Clinical characteristics of people with diabetes in NDR, NPDR and PDR group

Variable	NDR group (*N* = 172)	NPDR group (*N* = 184)	PDR group (*N* = 162)
SUA (μmol/L)	360.7 ± 100.2	397.4 ± 90.9^*^	421.5 ± 112.4^*#^
Homocysteine (μmol/L)	10.89 ± 4.56	22.56 ± 2.31^**^	30.77 ± 3.49^*#^
TC (mmol/L)	4.9 ± 1.65	6.2 ± 1.19^*^	6.9 ± 1.59^*^
LDL-c (mmol/L)	3.09 ± 0.89	3.55 ± 0.86^*^	3.78 ± 0.23^*^
Urinary albumin (mg/L)	16.0 ± 1.21	98.4 ± 1.46^**^	356.8 ± 4.69^**##^

^*^P < 0.05, ^**^P < 0.01 compared with people with diabetes in NDR group; ^#^*P* < 0.05, ^##^*P* < 0.01 compared with people with diabetes in NPDR group. *SUA* serum uric acid, *TC* total cholesterol, *LDL-c* low density lipoprotein cholesterol

### Risk factors for diabetic retinopathy

The associations of SUA, homocysteine, TC, LDL-c and urinary albumin levels with the severity of diabetic retinopathy were identified by using the multivariate logistic regression analysis. According to the results of multivariate logistic regression analysis, we identified that the severity of diabetic retinopathy was associated with high SUA level (odds ratio (OR) = 1.108; 95% confidence interval (CI) = 1.098–1.163; *P* = 0.032), high homocysteine level (OR = 1.021; 95% CI = 1.040–1.264; *P* = 0.041), high TC level (OR = 1.117; 95% CI = 1. 189–2.354; *P* = 0.037), high LDL-c level (OR = 1.026; 95% CI = 1.034–1.452; *P* = 0.026) and high urinary albumin level (OR = 1.003; 95% CI = 1.049–1.198; *P* = 0.043) (Table 3).

**Table 3:** Risk factors for diabetic retinopathy using multivariate logistic regression analysis

Parameters	OR	95% CI	*P*
SUA (μmol/L)	1.108	1.098–1.163	0.032
Homocysteine (μmol/L)	1.021	1.040–1.264	0.041
TC (mmol/L)	1.117	1.189–2.354	0.037
LDL-c (mmol/L)	1.026	1.034–1.452	0.026
Urinary albumin (mg/L)	1.003	1.049–1.198	0.043

*OR* odds ratios, *CI*: confidence interval, *SUA* serum uric acid, *TC* total cholesterol, *LDL-c* low density lipoprotein cholesterol

## Discussion

DR is a kind of serious microvascular complication of T2DM, causing visual impairment in adults. In our study, the included 518 individuals with T2DM were classified into NDR, NPDR, and PDR groups according to the severity of diabetic retinopathy. No significant difference in age, gender, BMI, and diabetes duration was found among these three groups. The multivariate logistic regression analysis showed that high SUA (OR = 1.108; 95% CI = 1.098–1.163; *P* = 0.032) and homocysteine (OR = 1.021; 95% CI = 1.040–1.264; *P* = 0.041) levels were associated with the severity of diabetic retinopathy. In the study of Srivastav et al., they firstly demonstrated that increased homocysteine levels were correlated with the reduction in retinal nerve fiber layer thickness and increased severity of diabetic retinopathy [18]. Liang et al. found that an increased SUA level was significantly correlated with the severity of albuminuria (OR, 1.227; 95% CI = 1.015–1.482; *P* = 0.034) and DR (OR, 1.264; 95% CI = 1.084–1.473; *P* = 0.003) in Taiwanese people with type 2 DM [19]. SUA concentration is associated with the increase in severity of DR over a 3-year period in people with T2DM [20]. Therefore, our results suggested that high SUA and homocysteine levels are risk factors of DR, which are consistent with some previous studies.

Leukocyte adhesion to the vascular endothelium is critical in the pathogenesis of DR, and this biological procedure is mediated by adhesion molecules, such as intercellular cell adhesion molecule-1 (ICAM-1) and monocyte chemoattractant protein-1 (MCP-1) [21, 22]. Meanwhile, interleukin-6 (IL-6), and tumor necrosis factor-alpha (TNF-α) are inflammatory factors also related to pathogenesis of DR [23, 24]. It has been reported that high uric acid can promote the expressions of adhesion molecules and inflammation mediators (including ICAM-1, MCP-1, IL-6, TNF-a) of the human retinal endothelial cells under high glucose, and increase the activity of Notch signaling pathway, suggesting high uric acid may promote the diabetic retinopathy by activating Notch signaling pathway [25]. In addition, the serum levels of ICAM-1 had been also confirmed to be associated with increased severity of diabetic retinopathy [26]. Therefore, SUA might play important roles in the severity of DR by influencing the signaling pathways related to inflammation and molecular adhesion.

In the study of Ahmad et al., the prevalence of microalbuminuria in people with T2DM is 31.56% and the microalbuminuria is identified as an early sign of diabetic nephropathy [27]. In our study, we found that high urinary albumin levels were associated with more serious diabetic retinopathy (OR = 1.003; 95% CI = 1.049–1.198; *P* = 0.043). Therefore, our results indicated that higher urinary albumin level is risk factor for the severity of DR in people with T2DM and has the potential to be used for early detection.

It has been reported that more serious endothelial cell damage might be caused in hypertensive subjects with microalbuminuria [28]. Studies also have shown that microalbuminuria is an independent risk factor for cardiovascular diseases not only in hypertensive and diabetic individuals but also for general population [29]. In the people with acute myocardial infarction, microalbuminuria is closely associated with functional disorder of endothelial progenitor cells, predicting the aggravation of coronary remodeling after percutaneous coronary intervention [30]. There is a positive correlation between microalbuminuria and the severity of coronary artery disease in T2DM individuals [15]. Besides, microalbuminuria is also an important tool for predicting the mortality and morbidity in individuals with cardiovascular and peripheral vascular diseases [31]. Therefore, we speculated that the microalbuminuria might participate in the DR by causing damage to the vascular endothelial cell. However, there are some limitations in our study. The diabetes treatment was not considered in our study. Besides, our study only included 518 individuals with T2DM during 2 years and classified into three groups according diabetic retinopathy severity. Thus, more studies with larger sample size or for people from different regions are still needed to validate the results of our study.

## Conclusion

In conclusion, we found that that high SUA, homocysteine, TC, LDL-c, and urinary albumin levels were associated with the severity of diabetic retinopathy. Higher SUA and urinary albumin levels could be considered as risk factors for severity of DR in individuals with T2DM.

## Data Availability

The datasets used and analysed during the current study available from the corresponding author on reasonable request.

## Abbreviations

DR: Diabetic retinopathy
T2DM: Type 2 diabetes mellitus
NDR: Non-diabetic retinopathy
NPDR: Non proliferative diabetic retinopathy
SUA: Serum uric acid
BMI: Body mass index
LDL-c: Low density lipoprotein cholesterol
TC: Total cholesterol
PDR: Proliferative diabetic retinopathy
